# Survival Outcomes and Pattern of Relapse After SABR for Oligometastatic Prostate Cancer

**DOI:** 10.3389/fonc.2022.863609

**Published:** 2022-04-14

**Authors:** Carole Mercier, Marc Claessens, Bart De Troyer, Tibaut Debacker, Karen Fransis, Hendrik Vandeursen, Piet Ost, Piet Dirix

**Affiliations:** ^1^ Department of Radiotherapy, Iridium Netwerk, Antwerp, Belgium; ^2^ Integrated Personalised and Precision Oncology Network, University Antwerp, Antwerp, Belgium; ^3^ Department of Urology, AZ KLINA, Brasschaat, Belgium; ^4^ Department of Urology, AZ Nikolaas, Sint-Niklaas, Belgium; ^5^ Department of Urology, ZNA Middelheim, Antwerp, Belgium; ^6^ Department of Urology, UZA, Edegem, Belgium; ^7^ Department of Urology, GZA Sint-Augustinus, Antwerp, Belgium; ^8^ Department of Human Structure and Repair, Ghent University, Ghent, Belgium

**Keywords:** oligometastasis, stereotactic ablative radiotherapy, stereotactic body radiotherapy, radiosurgery, prostate cancer, prostatic neoplasms, neoplasm recurrence, neoplasm metastasis

## Abstract

**Introduction:**

The addition of stereotactic ablative radiotherapy (SABR) to standard of care for patients with oligometastatic prostate cancer has the potential of improving survival and delaying further metastases. The primary aim of this analysis is to report survival outcomes and pattern of recurrence of patients with hormone-sensitive (HSPC) and castrate-resistant (CRPC) oligometastatic prostate cancer treated with SABR.

**Methods:**

This is a single-center retrospective study of patients with oligometastatic prostate cancer treated in Iridium Network between 2014 and 2018. All patients with oligometastatic (≤3 active lesions) HSPC and CRPC treated with SABR were included. Data were collected using electronic records. Patterns of first progression following SABR were reported. Kaplan-Meier methods were used to determine survival outcomes.

**Results:**

Eighty-seven men received SABR to 115 metastases. Nineteen patients were castrate-resistant and 68 hormone-sensitive at the time of SABR. Median follow-up was 41.6 months. In 25% of patients, no decline from baseline PSA was recorded. Median bPFS was 11.7 months (95% CI 7.6 - 18.3) for HSPC as well as CRPC (95% CI 6.4 - 24.0) (p=0.27). Median DMFS was 21.8 (95% CI 16.9 - 43.2) versus 17.6 months (95% CI 6.7 - 26.2) for HSPC versus CRPC, respectively (p=0.018). Median OS was 72.6 months (95% CI 72.6 – not reached) for HSPC and not reached for CRPC (95% CI 35.4 months – not reached) (p=0.026). For the subgroup of oligorecurrent HSPC, short-term androgen-deprivation therapy was associated with improved bPFS (median 6.0 vs. 18.3 months, HR 0.31, p<0.001) and DMFS (median 15.8 vs 29.6 months, HR 0.5, p=0.06). Information on pattern of relapse was retrieved for 79 patients: 45% (36/79) of these patients were long-term disease-free (>18 months), 28% (22/79) of patients wmere oligoprogressive (≤3 new lesions) and 27% (21/79) developed a polymetastatic relapse.

**Conclusion:**

In this cohort, oligometastatic HSPC showed potential benefit from SABR with a median DMFS of 21.8 months. Well-selected patients with oligometastatic CRPC may also benefit from SABR. For patients with metachronous and repeat oligorecurrent HSPC, combining SABR with short-term androgen-deprivation therapy was associated with improved bPFS and DMFS. Overall, 36/87 (41%) of patients were still free from clinical relapse at 18 months.

## Introduction

Oligometastatic disease (OMD) is defined as an intermediate stage of cancer spread between locoregional and widespread metastatic disease and can include a wide spectrum of disease biologies and clinical behaviors (1). OMD is increasingly diagnosed in prostate cancer (PCa) owing on the one hand to improved detection with advanced imaging like prostate-specific membrane antigen (PSMA) PET-CT and on the other hand to the success of systemic therapies in prolonging cancer survival. Even so, the biological features of OMD are poorly defined. Until biomarkers are identified to distinguish OMDs with truly limited metastatic capacity from those with fast-progressing behavior, it is a reasonable strategy to select patients based on clinical assumptions. The European Society for Radiotherapy and Oncology (ESTRO) and European Organisation for Research and Treatment of Cancer (EORTC) have proposed a classification in nine OMD subtypes, reflecting the different clinical states and underlying biological processes of OMD (2).

There is increasing evidence to suggest that patients with prostate OMD could benefit from more aggressive local treatment of the metastases, so-called metastasis-directed treatment, to obtain deep remission or possibly cure while preserving functional status (3, 4). In this regard, stereotactic ablative radiotherapy (SABR) offers a safe and effective treatment option.

Considering the lack of a standardized definition of oligometastatic disease, patient selection for SABR needs to be clarified. Therefore, the primary aim of this analysis was to report survival outcomes of a heterogeneous group of patients from a real-world setting with hormone-sensitive (HSPC) and castrate-resistant (CRPC) oligometastatic prostate cancer treated with SABR and analyze pattern of relapse.

## Materials And Methods

### Study Population

The current analysis is based on a single-center retrospective database of patients with oligometastatic PCa treated in Iridium Network between December 2014 and December 2018. All patients with oligometastatic (≤3 active lesions) HSPC and CRPC treated with SABR were included. Metastatic lesions could be diagnosed on conventional (bone scintigraphy or CT) or innovative (whole body magnetic resonance imaging; choline or PSMA PET-CT) imaging techniques. The analysis was approved by the Ethics committee of GZA Hospitals on 30 March 2021.

### Treatment

Technical aspects of SABR delivery for spinal, bone and lymph node metastases in our center have been previously described in detail (5, 6). Briefly, patients were simulated with CT scan in a comfortable, stable, and reproducible supine position. Gross tumor volume (GTV) was delineated on CT using all relevant co-registered diagnostic imaging. For spinal lesions, a clinical target volume (CTV) was delineated following the international consensus guideline (7). For other locations, CTV was equal to GTV and an isotropic margin of 3-5 mm, depending on disease site and dimensions, was added to CTV to obtain the planning target volume (PTV). Patients were treated with volumetric modulated arc therapy technique. Patient’s position was evaluated daily with cone-beam CT imaging before each treatment session. Optical surface monitoring was applied during patient set-up and treatment delivery.

A risk-adapted approach was used for dose-prescription, with targets located near organs at risk receiving a more fractionated treatment. Single, 3- and 5 fraction schedules were applied. Dose per fraction ranged from 5.0 to 20.0 Gy. The use of a short-course (i.e., 6 months) of androgen-deprivation therapy, given concurrently with SABR, was always suggested but was never mandatory. For CRPC patients, the current systemic treatment was generally continued during and after SABR, until further progression.

### Endpoint Assessment

Follow-up was typically scheduled every 3 months for the first 2 years following SABR and every 6 months from then on. Clinical examination and PSA values were obtained for every visit, while diagnostic imaging was planned according to physician choice.

Data were collected using electronic records. Best PSA response, biochemical progression-free survival (bPFS), distant metastasis-free survival (DMFS), overall survival (OS), local control of treated metastases (LC), as well as recurrence pattern were analyzed as endpoints. Survival endpoints were calculated from start of SABR to last follow-up or the occurrence of an event. For patients who had undergone radical prostatectomy, biochemical failure was defined as a PSA rise to 0.2 ng/mL from nadir after SABR or, if PSA did not nadir below 0.2 ng/mL, the first rise in PSA after reaching nadir. For patients treated with radiotherapy to the primary prostate, the Phoenix definition of biochemical progression was upheld, i.e., PSA nadir +2 ng/mL. Initiation of systemic therapy, local recurrence or distant recurrence prior to reaching numerical definition of PSA failure was considered as biochemical failure in both instances. Distant metastasis was defined as a new, metastatic lesion outside the SABR target volumes. Survival was defined as death from any cause. Local control was defined as the absence of radiological tumor growth within the irradiated region. Regarding recurrence pattern, there were 3 categories: long-term (>18 months) disease free, oligoprogressor (≤ 3 new lesions) and polymetastatic progressor (>3 new lesions).

### Statistical Analysis

Median follow-up was calculated using the reverse Kaplan-Meier method. Kaplan-Meier survivor function and log rank test were used to calculate time-to-event outcomes. Univariate analysis was performed to evaluate the association between clinical factors and survival with the log-rank test, and Cox proportional hazards regression was used to estimate hazard ratios (HR).

## Results

### Patient and Treatment Characteristics

We identified 87 patients receiving SABR to a total of 115 metastases. Patient, tumor and treatment characteristics are depicted in **Table 1**. The median PSA level at the time of SABR was 2.8 ng/mL; 19 patients (22%) were castrate-resistant and 68 (78%) hormone-sensitive at that time. Most patients (60/87, 69%) presented with a single metastasis, only three (3%) patients were treated for 3 lesions. Two patients with 2 metastases were treated with SABR for their spinal lesion and a moderate hypofractionated regimen for their non-spinal bone lesion. Of the 87 patients, 13 (15%) had pelvic lymph nodes, 7 (8%) had presence of M1 nodes, 63 (72%) had bone-only disease and 4 had both lymph node and bone metastases. Following the ESTRO/EORTC consensus recommendation of OMD (2), patients were classified as follows: 6/87 (7%) with synchronous OMD and 66/87 (76%) with metachronous OMD of which 52/87 (60%) metachronous oligorecurrence and 14/87 (16%) metachronous oligoprogression; 10/87 (11%) repeat OMD of which 8/87 (9%) repeat oligorecurrence and 2% repeat oligoprogression. There were 5 (6%) patients with polymetastatic disease having oligoprogression (4/87, 5%) or oligopersistence (1%).

**Table 1:** Patient, tumor and treatment characteristics.

Variable		n	%
Age (yr.)	Median [IQR]	69	[63 - 77]
Time from primary treatment (yr.)	Median [IQR]	4.5	[2.6 - 7.5]
ISUP Grade	1	17	20%
	2	18	21%
	3	17	20%
	4	20	23%
	5	13	15%
	NA	2	2%
Primary treatment modality	Surgery	52	60%
	Radiotherapy	28	32%
	Other^a^	7	8%
PSA at SABR (ng/mL)	Median [IQR]	2.8	[0.9 - 6.2]
ESTRO-EORTC classification	*De novo* synchronous OMD	6	7%
	Metachronous oligorecurrence	52	60%
	Metachronous oligoprogression^b^	14	16%
	Repeat oligorecurrence	8	9%
	Repeat oligoprogression	2	2%
	Induced OMD	5	6%
Androgen deprivation status	Hormone-sensitive	68	78%
	Castrate-resistant	19	22%
Concurrent hormonal therapy			
HSPC	ADT ≤ 6 months	19	22%
	ADT ≥ 2 year	13	15%
	Anti-androgen monotherapy	5	6%
	None	31	36%
CRPC	ADT	13	15%
	ADT + ARTA	6	7%
Number of lesions	1	60	69%
	2^c^	24	28%
	3	3	3%
Type of lesion	Lymph node (N1)	13	15%
	Lymph node (M1a)	7	8%
	Bone^d^ (M1b)	67	77%
Imaging (n=115)	Conventional	22	19%
	PSMA or choline PET-CT	89	77%
	whole body MRI	4	3%
Nr of fractions (n=115)	1	23	20%
	3	68	59%
	5	24	21%
Biologically Effective Dose (Gy)	Median [IQR]	230	[189 - 230]

a hormonal therapy, chemotherapy, High Intensity Focused Ultrasound.

b 2 patients were progressive under treatment with anti-androgen monotherapy; the others received androgen-deprivation therapy (ADT).

c two patients with 2 metastases were treated with SABR for their spinal lesion and a moderate hypofractionated regimen for their non-spine bone lesion.

d 4 patients had bone as well as lymph node metastases.

ADT, androgen-deprivation therapy; ARTA, androgen receptor-targeted agents; CRPC, castrate-resistant prostate cancer; HSPC, hormone-sensitive prostate cancer; ISUP, International Society of Urological Pathology; OMD, oligometastatic disease; PSA, prostate-specific antigen; PSMA, prostate-specific membrane antigen; SABR, stereotactic ablative radiotherapy.

Of the 68 patients (78%) who were hormone-sensitive, there were 31 patients (36%) who refused hormonal therapy concurrent with SABR. Of the other 37 HSPC patients, 19 patients were on androgen-deprivation therapy (ADT) for ≤6 months, five were on anti-androgen monotherapy, and 13 remained on ADT for at least 2 years. Eighty-nine of the 115 metastases (77%) were detected by PSMA or choline PET-CT, and 4 patients (3%) were staged with whole-body MRI. The remaining 22 lesions (19%) were detected on conventional CT and bone scintigraphy.

Most lesions (68/115, 59%) were treated with a 3-fraction schedule with a median dose of 10 Gy per fraction (range 6-10). Other commonly used fractionation schedules were a single fraction of 20 Gy (used for 23/115 lesions, 20%) and 5 fractions with a median dose of 7 Gy per fraction (range 5-7). Median biological effective dose (BED), calculated assuming an α/β-ratio of 1.5 Gy for prostate carcinoma using the linear quadratic model, was 230 Gy (8).

### PSA Response

**Figure 1:**
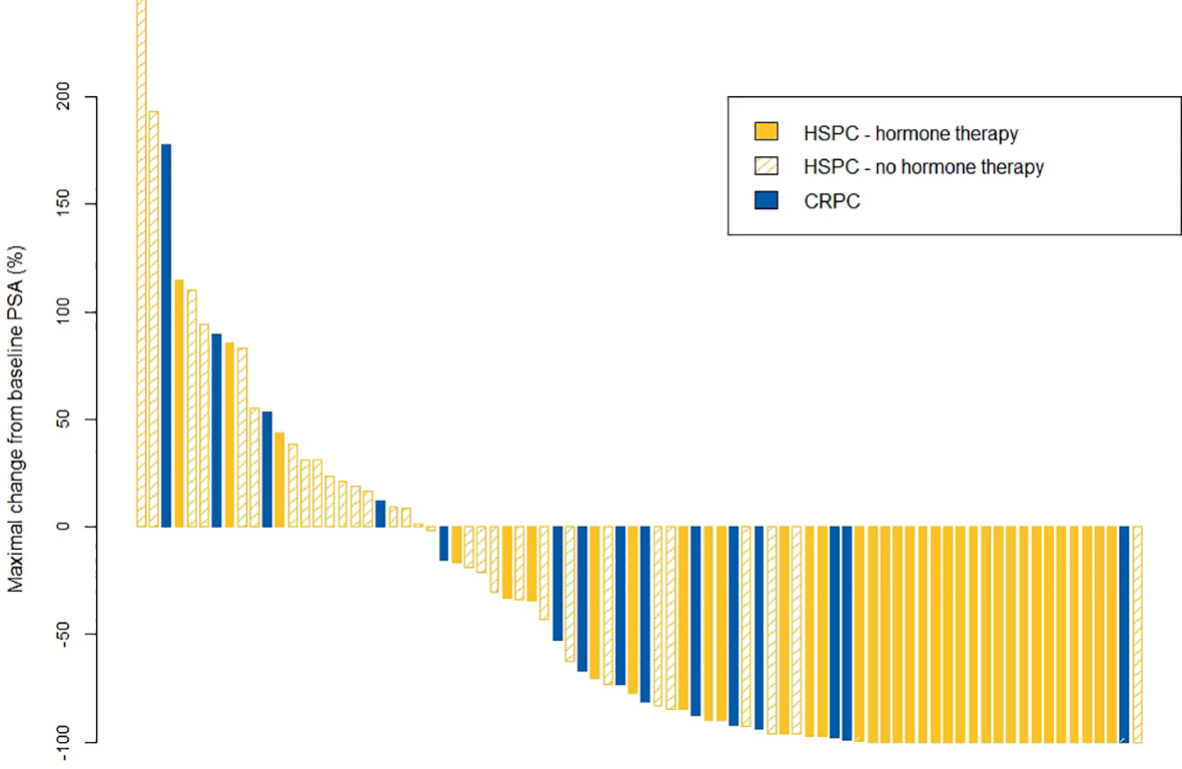
Waterfall plot for maximal changes in PSA value. CRPC, castrate-resistant prostate cancer; HSPC, hormone-sensitive prostate cancer; PSA, prostate-specific antigen.

**Figure 1** depicts the maximum change in PSA from baseline. In 26% of patients (23/87), no decline from baseline PSA was recorded. Of the patients without a PSA decline, four had castration-resistant disease (5%). Regarding oligorecurrent mHSPC patients, PSA declined in 27/29 (93%) patients receiving SABR + hormone therapy in comparison with only in 14/31 (45%) patients receiving SABR only.

### Survival Outcomes

**Figure 2:**
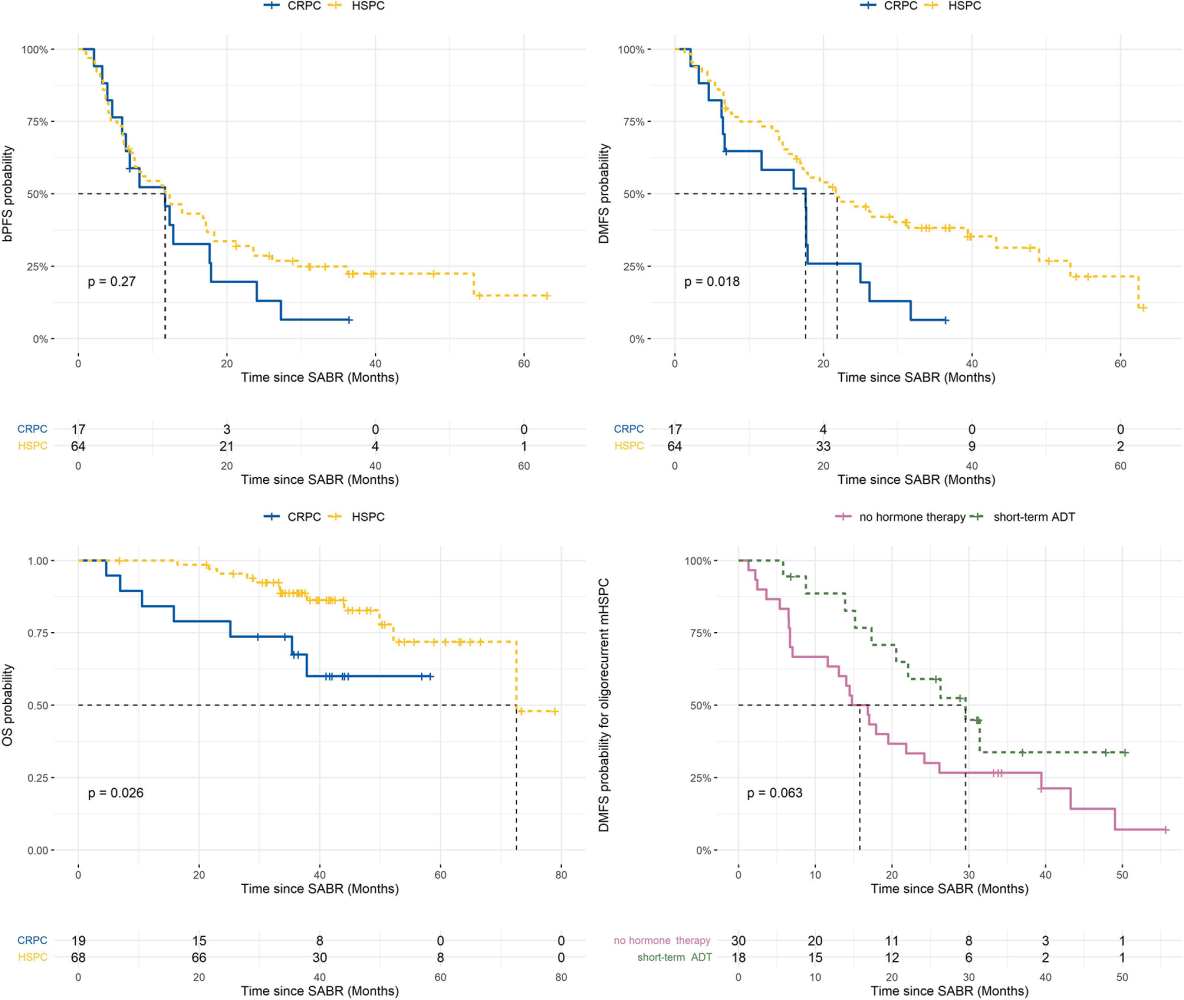
Kaplan-Meier plots of survival outcomes (9). ADT, androgen deprivation therapy; BPFS, biochemical progression-free survival; CRPC, castrate-resistant prostate cancer; DMFS, distant metastasis-free survival; HSPC, hormone-sensitive prostate cancer; OS, overall survival; SABR, stereotactic ablative radiotherapy. Risk tables are presented under the survival curves.

Median follow up time was 41. 6 months (IQR 35.7 - 50.8). Median bPFS was 11.7 months (95% CI 7.6 - 18.3) for HSPC with a 3-year rate of 25% (95% CI 16 - 39) compared to 11.7 months (95% CI 6.4 - 24.0) and 6% (95% CI 1 - 43) for CRPC, respectively (p=0.27). Median DMFS was 21.8 months (95% CI 16.9 - 43.2) with a 3-year rate of 38% (95% CI 28 - 53) in the HSPC cohort, compared to 17.6 months (95% CI 6.7 - 26.2) and 6% (95% CI 1 - 43) for CRPC, respectively (p=0.018). A median OS of 72.6 months (95% CI 72.6 – not reached) and 3-year rate of 89% (95% CI 81 - 97) was observed in the HSPC group; median OS was not reached (95% CI 35.4 months – not reached) and 3-year OS rate was 68% (95% CI 49 - 93) in the CRPC group (p=0.026). Kaplan-Meier survival curves for bPFS, DMFS and OS are shown in **Figure 2**.

For the subgroup of metachronous and repeat oligorecurrent metastatic HSPC (mHSPC), the impact of the addition of ≤6 months ADT to SABR on bPFS and DMFS was evaluated. On univariate analysis, short-term ADT was associated with improved bPFS (median 6.0 vs. 18.3 months, HR 0.31, p<0.001) and DMFS (median 15.8 vs 29.6 months, HR 0.5, p=0.06; **Figure 2**).

### Pattern of Relapse

Information on pattern of first progression after SABR could be retrieved for 79 patients. Patients were categorized within 3 categories: long-term (>18 months) disease free, oligoprogressor (≤ 3 new lesions) and polymetastatic progressor (>3 new lesions): 45% (36/79) of the evaluable patients did not develop a new recurrence within 18 months, 28% (22/79) of patients were oligoprogressive (≤3 new lesions) at first recurrence and 27% (21/79) developed a polymetastatic relapse (**Table 2**). Overall, 36/87 (41%) of patients were still free from clinical relapse at 18 months.

**Table 2:** Pattern of first progression after SABR.

	n	%
Long-term disease free (> 18 months)	36	41%
Oligoprogressive	22	25%
Polymetastatic relapse	21	24%
NA (lost to follow-up)	8	9%

SABR, stereotactic ablative radiotherapy.

Local relapse in the treated metastasis was detected in 7 bony lesions (5 originating from HSPC, 2 from CRPC), having received a median BED of 198 Gy (IQR 152 – 230). If local relapse occurred, it was detected after a median time of 38 months (IQR 22 – 57). LC rates at 1 year and 3 years were 100% and 96%, respectively.

## Discussion

In this article, we describe a single-institution experience treating PCa OMD with SABR at a median follow-up of 41.6 months. Our cohort consisted of 87 patients, a mixture of metachronous as well as synchronous OMD, HSPC as well as CRPC, oligorecurrent as well as oligoprogressive mPCa. In total, 41% of patients remained free from new metastases for a period of >18 months, and 25% of patients developed limited new metastases potentially amenable for repeat MDT within the first 18 months following SABR.

As a subgroup analysis, PSA response, bPFS and DMFS in (metachronous as well as repeat) oligorecurrent HSPC was evaluated. Two phase II RCT’s compared SABR to surveillance in the setting of metachronous oligorecurrent HSPC detected on choline PET-CT (3) or conventional CT and bone scintigraphy (4, 10), showing an advantage for MDT in terms of ADT-free survival and PFS. Despite 77% of our patients having PSMA PET-selected OMD, a more sensitive selection method than conventional CT, bone scintigraphy or choline PET-CT (11), our data do not compare as favorable as those reported in the aforementioned trials. It is remarkable that in our analysis, a PSA decline was measured in only 45% of patients receiving SABR without additional systemic treatment, as opposed to 75% in STOMP; median bPFS was 6 months in our cohort as opposed to >24 months in ORIOLE. These observations are probably related to several reasons, such as different bPFS definitions or the inclusion of 8 repeat oligorecurrent HSPC’s in our cohort.

However, it seems that still some cases were labelled as OMD if in fact it was only the tip of the iceberg for a subclinical polymetastatic disease. Therefore, we evaluated the impact of adding short-term ADT to SABR, to see if it is possible to eliminate potential micrometastases that are not (yet) visible and enhance the therapeutic effect. In our cohort, the addition of ADT for oligorecurrent HSPC was associated with improved bPFS (median 6.0 vs. 18.3 months, HR 0.31, p<0.001) and DMFS (median 15.8 vs 29.6 months, HR 0.5, p=0.06). While this analysis is exploratory and only hypothesis-generating, several phase II-III trials are currently testing the combination of SABR with either short course ADT (12–14) or androgen receptor pathway inhibitors (15). The main goal of this approach remains to postpone the start of lifelong ADT. Adding temporary hormonal treatment has already demonstrated improved LC, OS and MFS in primary treatment of high risk PCa (10).

New biomarkers may be critically important to help determining the natural history of the disease and to select the patients who could actually benefit from MDT. As of now, treatment decisions for OMD are based on clinical parameters such as number of metastases, time to recurrence and PSA doubling time. In a study by Deek et al., the presence of high-risk mutations was an independent prognostic factor allowing identification of patients who need more aggressive approaches beyond metastases-directed therapy (16). This suggests that tumor mutational profiles can provide a biological definition of OMD and complement currently used numerical definitions. Strategies for improved candidate selection have already been incorporated in prospective trials to provide a deeper understanding of the predictive role of biomarkers (12–14).

Looking at the oligo-CRPC patients of our analysis, DMFS and OS were significantly shorter compared to the HSPC patients, owing to the more advanced disease stage. Despite the more aggressive setting, median DMFS was still 17.6 months. During this time interval, it was not necessary to switch to a next line of systemic therapy. Moreover, in-field control was excellent, confirming the radiosensitivity of CRPC and the high efficacy of SABR on local metastatic control. While retrospective analyses testing the addition of SABR report encouraging results with DMFS ranging between 11 – 12 months (17–19), prospective data on the use of SABR in oligometastatic CRPC remain scarce, and several questions remain to be answered. For example, should we rather add SABR to the mainline systemic treatment to postpone next systemic treatment, or radically treat the visible metastases using SABR in combination with a switch to a next type of systemic treatment to target macroscopic as well as microscopic treatment-resistant disease? Regarding outcomes, is PFS a meaningful endpoint, or should we enroll larger patient groups and maintain long follow-up periods to evaluate OS endpoints? These questions warrant further exploration in prospective trials using standardized treatments to validate the potential benefit and to define the group of CRPC patients expected to profit from SABR. Ongoing prospective trials, such as the single-arm phase II TRAP trial (20) and the Medcare trial (21), are investigating the role of SABR for oligometastatic CRPC. Since immune-checkpoint inhibitor monotherapy has shown only modest benefits in the mCRPC setting, the ICE-PAC trial aimed at improving outcomes by combining the PD-L1 checkpoint inhibitor avelumab with SABR in patients with both low- as well as high-volume mCRPC after prior androgen-receptor pathway inhibitor therapy (22). A median radiographic PFS of 8.4 months was observed in this heavily pretreated patient group, of which the majority had >10 metastases.

The present study was limited by its retrospective nature, by the relatively small patient group and by the rather heterogeneous patient, imaging and tumor characteristics. The current cohort reflects a real-world representation of the imaging evolution in recurrent prostate cancer. Initially, conventional imaging was supplemented with whole body MRI before the advent of choline PET-CT and PSMA PET-CT improved the detection of oligometastatic disease. The results might have been even better when patients received the most sensitive imaging at restaging, as was also shown in ORIOLE where PFS and DMFS was improved when all PSMA-PET positive lesions were treated with SABR (4).

## Conclusion

In this cohort, patients with hormone-sensitive oligometastatic disease showed potential benefit from SABR with a median distant metastasis-free survival of 22 months. Well-selected patients with oligometastatic CRPC may also benefit from SABR. For patients with metachronous and repeat oligorecurrent HSPC, combining SABR with short-term androgen-deprivation therapy was associated with improved bPFS and DMFS. Overall, 36/87 (41%) of patients were still free from clinical relapse at 18 months.

## Data Availability Statement

The data that support the findings of this study are available upon request from the corresponding author, CM, carole.mercier@gza.be. The data are not publicly available due to their containing information that could compromise the privacy of patients.

## Ethics Statement

The studies involving human participants were reviewed and approved by Commissie Medische Ethiek GZA Ziekenhuizen, Oosterveldlaan 22, 2610 Wilrijk. Written informed consent for participation was not required for this study in accordance with the national legislation and the institutional requirements.

## Author Contributions

CM had full access to all the data in the study and takes responsibility for the integrity of the data and the accuracy of the data analysis. Study concept and design: CM, PD, and PO. Acquisition of data: CM and PD. Analysis and interpretation of data: CM, PD, and PO. Drafting of the manuscript: CM, MC, BT, TD, KF, HV, PO, and PD. Critical revision of the manuscript for important intellectual content: CM, MC, BT, TD, KF, HV, PO, and PD. Statistical analysis: CM, PD, and PO. Supervision: CM, PD, and PO. All authors contributed to the article and approved the submitted version.

## Conflict of Interest

The authors declare that the research was conducted in the absence of any commercial or financial relationships that could be construed as a potential conflict of interest.

## Publisher’s Note

All claims expressed in this article are solely those of the authors and do not necessarily represent those of their affiliated organizations, or those of the publisher, the editors and the reviewers. Any product that may be evaluated in this article, or claim that may be made by its manufacturer, is not guaranteed or endorsed by the publisher.
